# Carbon nanotubes: a promising tissue engineering approach for *in vitro* cultivation & differentiation of primary canine articular chondrocytes

**DOI:** 10.1186/1471-2474-16-S1-S20

**Published:** 2015-12-01

**Authors:** Brigitta Matta-Domjan, Alice King, Mazhar Ajaz, Csaba Matta, Rebecca Lewis, Hugo Macedo, Roberto La Ragione, Eirini Velliou, Alan Dalton

**Affiliations:** 1 Department of Chemical & Process Engineering, Faculty of Engineering & Physical Sciences, University of Surrey, Guildford, GU2 7XH, UK; 2 Department of Physics, of Surrey, Faculty of Engineering & Physical Sciences, University of Surrey, Guildford, GU2 7XH, UK; 3 Department of Microbial and Cellular Sciences, Faculty of Health and Medical Sciences, University of Surrey, Guildford, Surrey, GU2 7XH, UK; 4 Royal Surrey Country Hospital, NHS Foundation Trust, Guildford, Surrey, GU2 7 XX, UK; 5 Smart Separations Ltd, London, UK; 6 School of Veterinary Medicine, Faculty of Health and Medical Sciences, University of Surrey, Guildford, Surrey, GU2 7XH, UK

## 

Development of biocompatible materials has great potential in biomedical engineering both for *in vitro* studies as well as for *in vivo* applications. Two- and three-dimensional carbon nanotube (CNT) substrates imitating and providing an extracellular matrix-like structure are promising constructs as cell-supporting scaffolds. Lately, they have received considerable interest in tissue engineering; however, cellular responses to nanoscale stimuli need to be better understood.

Here, we present the preliminary results on the effect of CNT-based scaffolds on the proliferation and arrangement of primary canine chondrocytes (PCCs). We aim to develop scaffolding materials for the *in vitro* cultivation of normal and neoplastic cells with the ultimate objective of using them for applications such as tissue implants in cartilage repair and tissue regeneration after surgical intervention.

In the proposed studies we aim to use an aerogel network of CNTs that has been drawn from a vertically aligned array as a synthetic substrate for the growth and alignment of primary canine chondrocytes. This aerogel consists of CNTs that are aligned parallel to the major axis of the CNTs; they have exceptionally low densities, are electrically and thermally conductive whilst maintaining very high tensile strength and elasticity. We are studying the cell growth, adhesion, morphology, viability and metabolism of cells seeded onto CNT substrates.

Preliminary results to date have revealed that PCCs are capable of proliferating on CNT-based scaffolds, although the cell viability seems to be slightly decreased in comparison to the conventional 2D cell culture. Moreover, our nanosubstrates are able to induce directional cell growth of PCCs via aligning cells along CNTs. The latter is essential for *in vivo* application of nanosubstrates in tissue regeneration.

